# The effects of the COVID-19 pandemic on pediatric dog bite injuries

**DOI:** 10.1186/s40621-024-00537-x

**Published:** 2024-10-03

**Authors:** Paul T. Menk, E. Melinda Mahabee-Gittens, Wendy J. Pomerantz

**Affiliations:** 1 Division of Emergency Medicine, Children’s Healthcare of Atlanta, Atlanta, GA USA; 2 Department of Pediatrics, Emory University School of Medicine, Atlanta, GA USA; 3 Division of Emergency Medicine, Cincinnati Children’s Hospital Medical Center, 3333 Burnet Ave, Cincinnati, OH 45229 USA; 4 Department of Pediatrics, University of Cincinnati College of Medicine, Cincinnati, OH USA; 5 Pediatric Emergency Medicine, Children’s Healthcare of Atlanta, 1547 Clifton Road NE, 2nd Floor, Atlanta, GA 30322 USA

**Keywords:** Dog bites, Pediatric Injury, COVID-19 Pandemic

## Abstract

**Background/objective:**

Shortly after the onset of the COVID Pandemic, when many schools and outside activities were suspended, dog adoption rates increased. It is unknown if increased dog adoption rates along with stay-at-home orders resulted in changes to pediatric dog bite injuries. The objective of this study was to examine the incidence and characteristics of dog bites in children seen in a pediatric emergency department (PED) during the pandemic compared to before.

**Methods:**

A retrospective review of children evaluated in the PED of a level 1 pediatric trauma center and its satellite PED from March 2018 through February 2022 who had a discharge diagnosis of dog bite (ICD-10 W54.0XXA) was conducted. Pre-pandemic cases, March 2018 through February 2020, were compared to those that occurred during the pandemic, March 2020 through February 2022.

**Results:**

There were 2,222 patients included in the study. Compared to pre-pandemic cases, the incidence for the first 12 months of the pandemic was 1.5 times higher than the pre-pandemic 12-month periods but returned closer to the pre-pandemic rates during the second 12 months of the pandemic. More patients were admitted during the pandemic (6.1% vs. 3.7%, *p* < 0.05). Facial and multiple injuries occurred more frequently during the pandemic (face 35.9% vs. 33.5%: multiple 18.5% vs. 15.6% *p* < 0.05).

**Conclusions:**

There was a higher incidence of PED visits, higher admission rates, and an increase in multiple body part and facial injuries in children with dog bite injuries during the COVID pandemic compared to pre-pandemic. Pediatric providers should emphasize safe dog interactions with anticipatory guidance.

## **Introduction**

The COVID-19 pandemic brought significant turmoil to the United States and its healthcare system. Many adult hospitals were forced to board patients in their emergency departments (EDs) as their intensive care units became critically full. Outside of the hospital, school closures and stay-at-home orders altered people’s daily routines and their home dynamics. Initially during this disruption, many people and families became more interested in dog ownership. Based on Google trends, dog adoption-related searches during April 2020 were significantly higher compared to the previous 5-year average (Ho et al. 2021). Shelters reported a 9% annual increase in dog adoptions during 2020 as compared to 2019 (Shelter Animals Count 2020). While dog ownership can provide mental health benefits (Hui Gan et al. 2020; Oliva and Johnston 2021), it is not without risk. In the United States, animal bites/stings consistently rank among the top six causes of unintentional injuries in the pediatric population (Borse and Sleet 2008). There are approximately 140,000 annual ED visits for pediatric dog bites (Loder 2019). The potential increase in child-dog interactions during the COVID-19 pandemic due to stay-at-home orders and increased adoption rates only amplified this risk.

Data from the United States and globally shows that dog bite injuries in the pediatric population increased significantly during the COVID-19 pandemic (Plana et al. 2022; Parente et al. 2021; Tulloch et al. 2021; Dixon and Mistry 2020). However, current studies on pediatric dog bites during the COVID-19 pandemic focus mainly on the initial 6 to 10 months. Studies have shown that dog adoption related searches returned to their pre-pandemic levels by December of 2020 (Ho et al. 2021). While still lower than before the pandemic, the number of dogs returned by owner increased by 6.7% in 2021 compared to 2020 (Shelter Animals Count 2022). With these changes, it is unclear if the initial spike in dog bite injuries remained constant throughout the pandemic. This study examines the first 24 months of the pandemic to help understand the full effects it had on dog bite injuries in children. Specifically, the study objective was to examine the incidence and characteristics of dog bites in 0-18-year-old children seen in a pediatric emergency department (PED) during the COVID-19 pandemic compared to before the pandemic.

## **Methods**

A retrospective study was conducted using data from the trauma registry of a level 1 pediatric trauma center PED and its satellite PED that were in a midwestern tertiary care children’s hospital. Data was extracted on patients < 18 years old who were seen March 1, 2018 through February 28, 2022 who received the diagnosis of a dog bite (ICD W54.0XXA) during their PED visit. Patients were excluded if they: were > 18 years old, left without being seen, or had subsequent visits for the same injury. The following information was abstracted from PED records of eligible patients: demographic information (e.g., age, sex, race, ethnicity, insurance); injury characteristics - anatomic location; patient disposition - discharged home or admitted to the hospital, intensive care unit (ICU), or operating room (OR). The pre-pandemic and during pandemic groups were defined as patients seen from March 1, 2018, through February 29, 2020, and March 1, 2020, through February 28, 2022, respectively. For further delineation, March 2018 through February 2019 is referred to as the first 12 months of the pre-pandemic and March 2019 through February 2020 is the second 12 months. Similarly, March 2020 through February 2021 is referred to as the first 12 months of the pandemic and March 2021 through February 2022 is referred to as the second 12 months of the pandemic. Student’s t-test was used to compare continuous variables. Pearson chi-squared test was used to compare categorical variables. Significance was set at α < 0.05. All analysis was completed using IBM^®^ SPSS^®^ Statistics (Version 26). This study was deemed exempt by the institutional review board as the dataset contained only de-identified data.

## **Results**

**Fig. 1:**
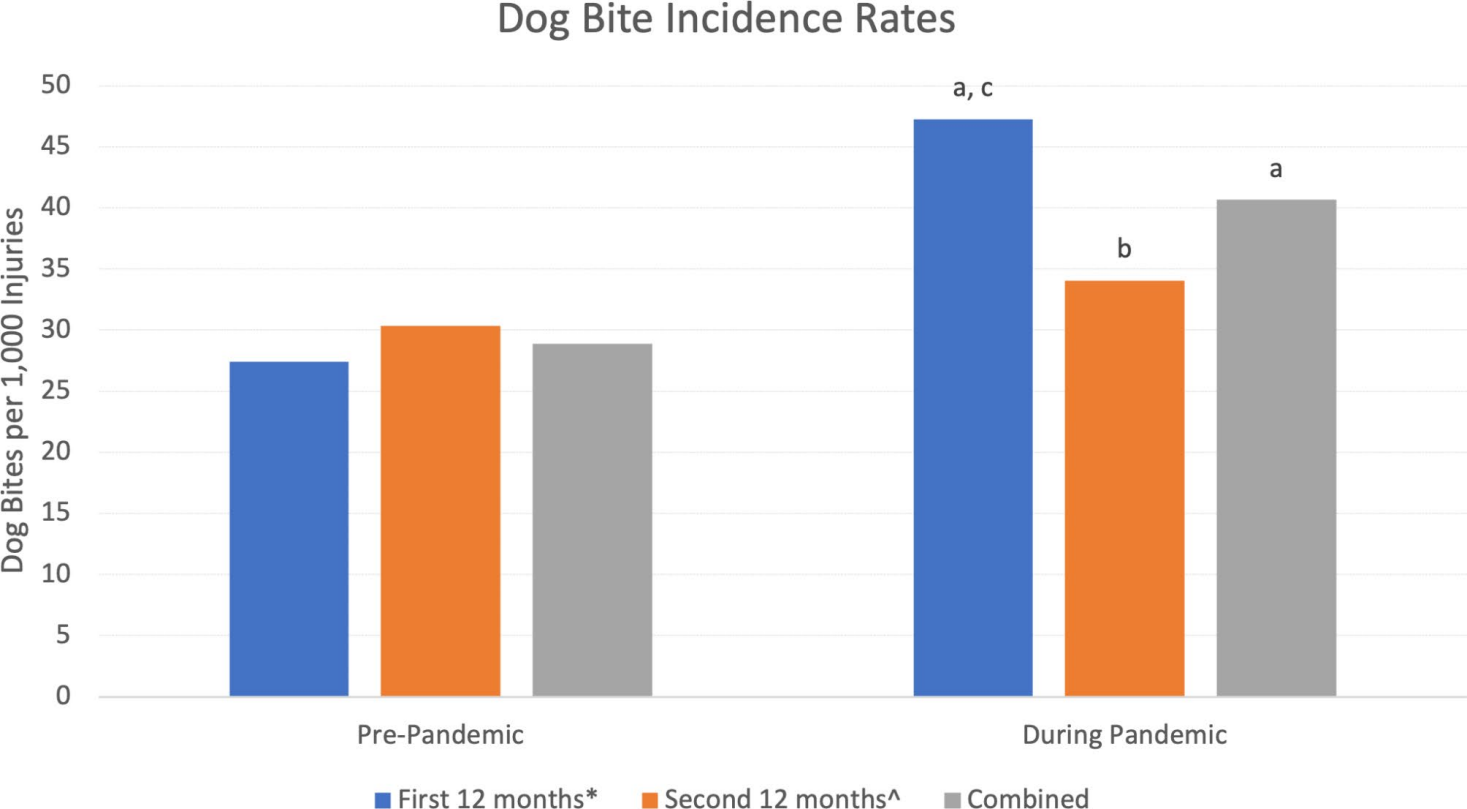
Dog bite incidence rates by year ^a^ Significantly higher than all pre-pandemic rates, *p* < 0.01 ^b^ Significantly higher than pre-pandemic first 12 months incidence rate, *p* < 0.01 ^c^ Significantly higher than the post-pandemic second 12 months, *p* < 0.01 ^*^ The first 12 months covered March 2018 through February 2019 for the pre-pandemic group and March 2020 through February 2021 for the post pandemic group ^^^ The second 12 months covered March 2019 through February 2020 for the pre-pandemic group and March 2021 through February 2022 for the post pandemic group

Of the 65,204 injury-related patients seen in the PED during the study period, 2,362 were due to dog bites (3.6%). Of these, 140 patients were excluded due to either age > 18 years (*n* = 37), left without being seen (*n* = 16), or subsequent visit for the same injury (*n* = 87); thus, 2,222 patients with dog bite injuries were eligible for analysis. Overall, there were 114 more cases during the COVID-19 pandemic (1,168) than prior to the pandemic (1,054). As noted in Fig. 1, the incidence rate of dog bite injuries was significantly higher during the first 12 months of the pandemic compared to (1) both years before the pandemic and (2) the second 12 months of the pandemic (for all comparisons, *p* < 0.001). However, the incidence rate during the second 12 months of the pandemic was only significantly higher than the first 12 months pre-pandemic (*p* < 0.01).

The mean (SD) age for the entire study sample was 7.64 (4.6) years; males sustained more dog bites than females (55.8% vs. 44.2%, Table 1). Although children aged 5 to 9 years old were the most affected group both before the pandemic and during (33.6% and 35.2% respectively), there were no statistically significant differences between age groups during the pre-pandemic years compared to the during pandemic years. Further, there were no differences based on child sex, race, or ethnicity. However, there was a higher number of patients who had private insurance during the pandemic compared to before the pandemic (60.2% vs. 49.8%, *p* < 0.001).

**Table 1:** Characteristics of dog bite injury patients before and during the COVID pandemic

	Pre-Pandemic Dog Bites *n* = 1054	During Pandemic Dog Bites *n* = 1168	*p*-value
Age, years (SD)	7.58 (4.6)	7.68 (4.6)	0.62
Male sex, n (%)	594 (56.4)	647 (55.4)	0.65
Age, years, n (%)			0.91
<1	23 (2.2)	27 (2.3)	
1–4	348 (33)	366 (31.3)	
5–9	354 (33.6)	411 (35.2)	
10–14	243 (23.1)	272 (23.3)	
15–18	86 (8.1)	92 (7.9)	
Race, n (%)			0.25
Black	242 (23.3)	228 (19.7)	
White	750 (72)	869 (75.4)	
Other^1^	49 (4.7)	56 (4.9)	
Hispanic ethnicity, n (%)	52 (5)	63 (5.4)	0.62
Insurance, n (%)			**< 0.001**
Private	518 (49.8)	612 (60.2)	
Government	448 (43.1)	343 (33.8)	
Self-pay	74 (7.1)	11 (6)	
Admissions^2^, n (%)	22 (2.1)	71 (3.8)	**0.02**
Required Operative Management^3^, n (%)	32 (3)	57 (4.9)	**0.03**

*p*-values in bold are statistically significant

^1^ Defined as American Indian, Asian, Middle Eastern, or Other

^2^ Defined as admitted to the intensive care unit or general wards

^3^ Includes patients directly admitted from the PED to operating room and patients admitted to other units that subsequently required operative management

**Fig. 2:**
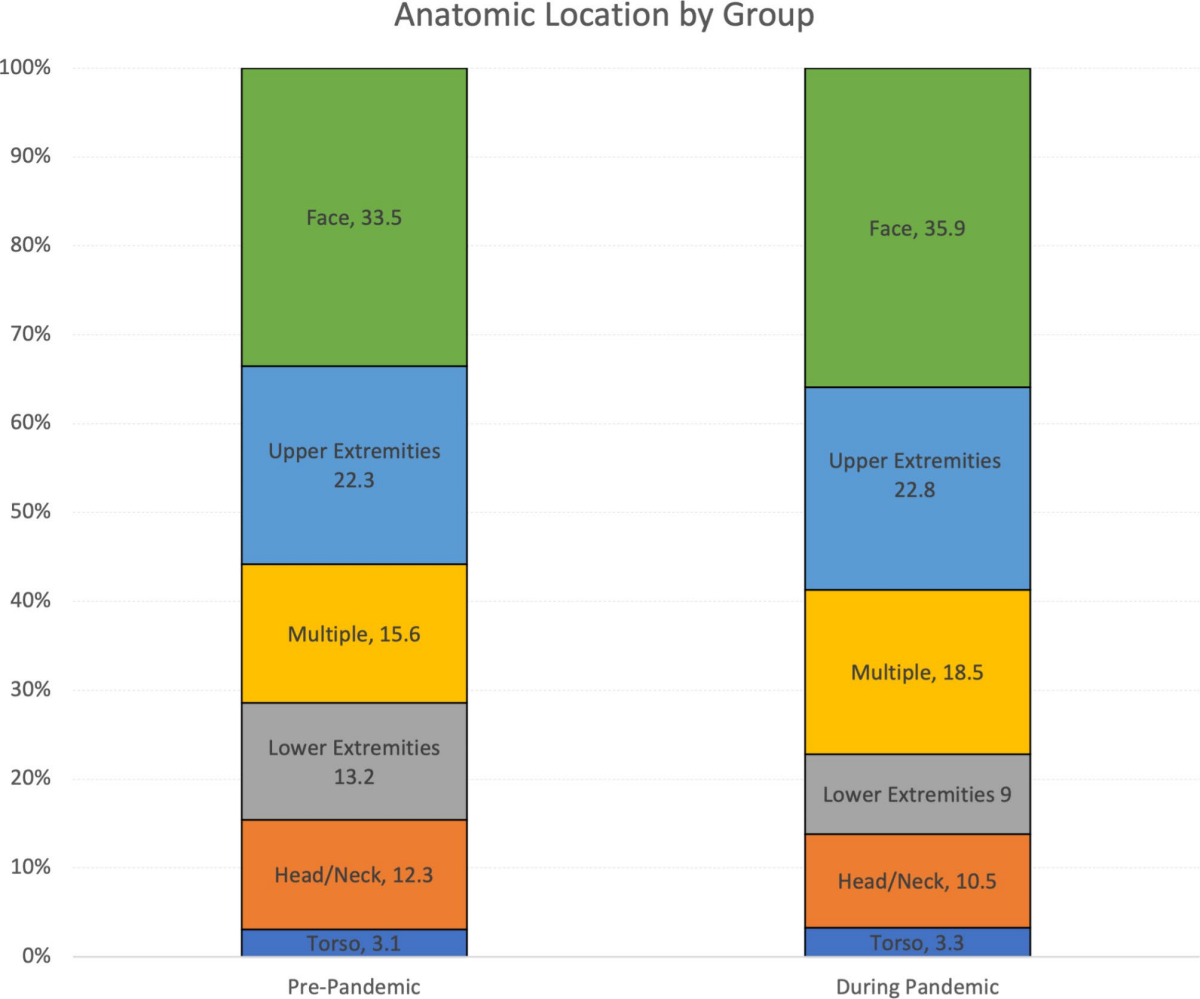
Anatomic location of dog bite by pandemic group

More patients required admission for their injuries during the pandemic compared to prior (3.8% vs. 2.1%, *p* = 0.02). Additionally, more patients required operative management during the pandemic than prior to the pandemic (4.9% vs. 3%, *p* = 0.03). During the pandemic, there was a significantly higher proportion of patients that sustained facial injuries and injuries to multiple body parts compared to before the pandemic (face: 35.9% vs. 33.5%, respectively; multiple body parts: 18.5% vs. 15.6%, respectively; all *p* < 0.001; see Fig. 2).

## **Discussion**

This study provides further evidence that there was a higher incidence rate as well as an absolute increase in dog bite injuries during the COVID-19 pandemic. The increase in dog adoption rates coupled with children spending more time at home due to school closures, activity cancellations, and stay-at-home orders likely contributed to this. These increased child-dog interactions may continue to persist as more people are continuing to work from home, even though the United States has relaxed COVID-19-related restrictions (Parker et al. 2022).

When examined by year, the incidence rate of dog bites for the first 12 months of the pandemic was significantly higher than the second 12 months. While a direct correlation cannot be identified, this does coincide with the gradual return to normalcy. In Ohio, the resident state for the hospital in this study, as state health orders for mandated masking and capacity limits ended on June 2nd, 2021 (Planalp 2021). The percent of Ohio public schools delivering 5-day in person learning rose from 41% at the start of the 2020–2021 school year to 78% at the end of the school year (Burbio 2023).

The COVID-19 pandemic did not change the injury trends for age groups, sex, race, or ethnicity, in our population. However, significantly more visits were made by children with private insurance during the pandemic than before. This finding contradicts a previous report that showed more dog bites occurring in households with government funded insurance (Plana et al. 2022). A possible reason for this is that those who were able to afford private insurance also had the fiscal ability to cover the costs of adopting and caring for a dog during the pandemic. As private insurance is often a marker of higher socioeconomic status, another reason may be that these families had fewer barriers, such as transportation, to obtaining medical care for dog bites.

Compared to pre-pandemic, during the pandemic, there was a statistically higher number of patients who: were admitted (3.7% vs. 6.1%, respectively), needed surgical intervention (3% vs. 4.9%, respectively), sustained facial injuries (33.5% vs. 35.9%, respectively), and sustained multiple injuries 15.6% vs. 18.5%, respectively). Interestingly, this increase in admissions for dog bites occurred at a time when hospital admissions fell from 2.24% of all hospital encounters to 1.89% at the study hospital (Cincinnati Children’s Hospital Medical Center 2023). This finding along with more patients needing surgical intervention during the pandemic seems to suggest that more significant injuries occurred after the onset of the COVID-19 pandemic. This is further supported by the increase in facial and injuries to multiple body parts seen during the COVID-19 pandemic. However, it is unclear if these admissions and surgeries were due to the injury itself or due to complications. It is possible that due to the fear of contracting COVID-19, families delayed seeking treatment until wounds became infected, potentially increasing the need for surgical debridement or admission for intravenous antibiotics. Additionally, patients with minor injuries may have utilized telehealth instead of presenting to the PED. Further research is warranted to examine indicators that suggest that admission or surgical intervention is needed for dog bite related visits; these indicators may include time between presentation to the PED and when the dog bite occurred.

The observational aspect of this study presents limitations that should be considered. The reliance on diagnosis codes for inclusions potentially excludes patients that were miscoded. While the use of the trauma registry provided a standardized data set, it limited the ability of this study to examine variables that were not included in the registry such as antibiotic use, suture use, or dog breed; all of which should be studied in future work. As this study was a single center study, its generalizability to the rest of the U.S. is limited. Nonetheless, this study utilized a large sample size and equal time-period comparisons between groups to help understand the full effects the COVID pandemic had on dog bite injuries in the pediatric population.

## **Conclusion**

In conclusion, there was a higher absolute number and incidence rate of PED visits, higher admission rates, more privately insured patients, and an increase in facial and multiple body part injuries during the COVID pandemic compared to prior. These results highlight the need for improved dog bite prevention education. Providers who care for children should include anticipatory guidance related to ways in which children can safely interact with their dogs (e.g., no rough games) and circumstances under which children should avoid such interactions (e.g., when the dog is eating) (American Academy of Pediatrics 2018; American Veterinary Medical Association 2023). Further, parents should be advised that they should supervise all child-dog interactions, especially when their children are young.

## Data Availability

De-identified individual participant data will not be made available.

## Abbreviations

PED: Pediatric emergency department
ED: Emergency department
